# Retraction: Relaxin improves multiple markers of wound healing and ameliorates the disturbed healing pattern of genetically diabetic mice

**DOI:** 10.1042/CS-2013-0105_RET

**Published:** 2024-11-20

**Keywords:** angiogenesis, diabetes, matrix metalloproteinase (MMP), relaxin, vascular endothelial growth factor (VEGF), wound healing

This article “Relaxin improves multiple markers of wound healing and ameliorates the disturbed healing pattern of genetically diabetic mice” DOI: 10.1042/CS20130105 is being retracted from *Clinical Science* at the request of the Editor-in-Chief and the Editorial Board following receipt of a notification from a reader, alerting the Editorial Board to concerns surrounding the Western blot and immunohistochemistry data. Specifically:

- Figure 2 Phospho e-NOS, a part of which seems to appear as Figure 2B 5-Lox panel from Altavilla et al, 2012 (DOI: 10.1111/j.1476-5381.2012.01969.x) [retracted]
- Figure 2 eNOS panel which seems to appear in Figure 2B β-actin panel from Bitto et al, 2010 (DOI: 10.1016/j.jvs.2010.05.116)
- Figure 2 β-actin panel which seems to appear as Figure 2B β-actin panel from Altavilla et al, 2012 (DOI: 10.1111/j.1476-5381.2012.01969.x) [retracted]
- Figure 3E which resembles Figure 2F from Galeano et al, 2011 (DOI: 10.1016/j.bbadis.2011.03.012), purportedly from different treatment conditions.

The authors have been contacted with regards to the concerns raised. The authors state that due to the age of the paper, the original data for Figure 2 and Figure 3E are no longer available, but the original samples are available. However, given the concerns surrounding the data validity, labelling, and handling in this paper, the conclusions reported in the article are not considered reliable, and the Editorial Board stands by the decision to retract this article. The authors were contacted regarding the retraction and disagree with the journal's decision.

